# Polyclonality overcomes fitness barriers in *Apc*-driven tumorigenesis

**DOI:** 10.1038/s41586-024-08053-0

**Published:** 2024-10-30

**Authors:** Iannish D. Sadien, Sam Adler, Shenay Mehmed, Sasha Bailey, Ashley Sawle, Dominique-Laurent Couturier, Matthew Eldridge, David J. Adams, Richard Kemp, Filipe C. Lourenço, Douglas J. Winton

**Affiliations:** 1 Cancer Research UK Cambridge Institute, Li Ka Shing Centre, Cambridge, UK; 2 Tumour Cell Biology Laboratory, The Francis Crick Institute, London, UK; 3 Wellcome Sanger Institute, Wellcome Trust Genome Campus, Hinxton, Cambridge, UK

**Keywords:** Cancer stem cells, Cancer genetics, Colorectal cancer

## Abstract

Loss-of-function mutations in the tumour suppressor *APC* are an initial step in intestinal tumorigenesis^1,2^. *APC*-mutant intestinal stem cells outcompete their wild-type neighbours through the secretion of Wnt antagonists, which accelerates the fixation and subsequent rapid clonal expansion of mutants^3–5^. Reports of polyclonal intestinal tumours in human patients and mouse models appear at odds with this process^6,7^. Here we combine multicolour lineage tracing with chemical mutagenesis in mice to show that a large proportion of intestinal tumours have a multiancestral origin. Polyclonal tumours retain a structure comprising subclones with distinct *Apc* mutations and transcriptional states, driven predominantly by differences in KRAS and MYC signalling. These pathway-level changes are accompanied by profound differences in cancer stem cell phenotypes. Of note, these findings are confirmed by introducing an oncogenic *Kras* mutation that results in predominantly monoclonal tumour formation. Further, polyclonal tumours have accelerated growth dynamics, suggesting a link between polyclonality and tumour progression. Together, these findings demonstrate the role of interclonal interactions in promoting tumorigenesis through non-cell autonomous pathways that are dependent on the differential activation of oncogenic pathways between clones.

## Abstract (web summary)

Multicolour lineage tracing and mutagenesis studies in a mouse model show that many intestinal tumours are polyclonal, with multiple clones exhibiting independent *Apc* mutations driven by differences in KRAS and MYC signalling.

## Main

The earliest event in the initiation of colorectal cancer (CRC) is the fixation of cancer driver mutations within the colonic epithelium, a process that requires successful competition with wild-type intestinal stem cells^8,9^ (ISCs). Biased competition that favours fixation of loss-of-function of the tumour suppressor *APC* has recently been ascribed to ‘supercompetitor’ behaviour, comprising both cell-intrinsic behaviours and non-cell autonomous suppressive effects on wild-type ISC neighbours^3–5^. Secretion of Wnt antagonists such as NOTUM acts to suppress wild-type stem cells within the same and adjacent crypts. Suppression of wild-type ISCs therefore promotes both fixation and subsequent expansion of APC-deficient crypts.

Biased competition by neighbourhood suppression is consistent with the consensus view of the past fifty years that most cancers are clonal in origin and evolve through branching evolution^10–13^. Genetically engineered mouse models providing a tissue-wide ‘first hit’ of *Apc* either in the germline or somatically and requiring only a further single sporadic mutation of the wild-type allele for tumour initiation provide a simple and immediate route to clonal tumorigenesis. Paradoxically however, adenomas that arise in individuals with familial adenomatous polyposis (FAP) or sporadically are often polyclonal^6,14–16^. Mouse models similarly show polyclonality^7,17–21^. Here we set out to determine the extent and nature of the clonal interactions that define polyclonal tumour formation and to understand how they relate to supercompetitor behaviour.

## Confetti reveals tumour polyclonality

**Fig. 1:**
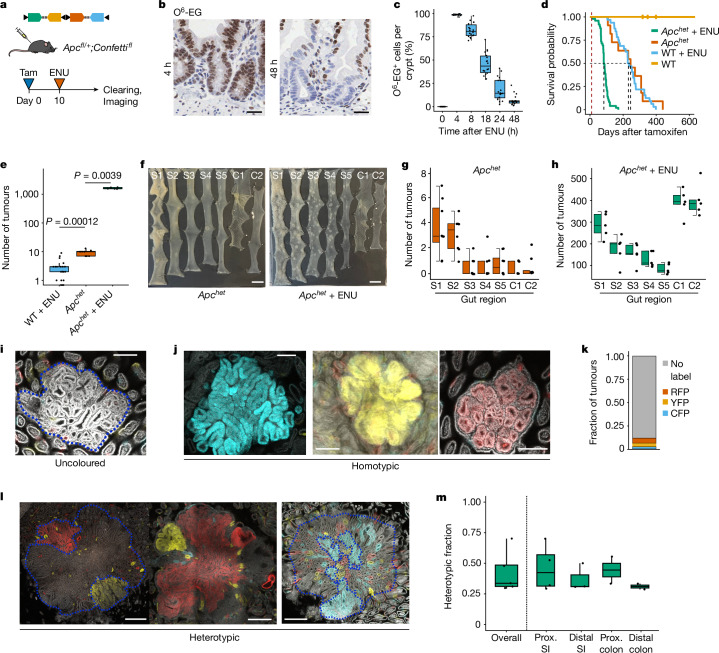
Heterotypic expression of Confetti identifies polyclonal origins of *Apc*-deficient tumours. **a**, Schematic of experimental approach. Tam, tamoxifen. **b**, Immunohistochemistry for *O*-6-ethyl-guanine (O^6^-EG) in small intestinal crypts after ENU treatment. Scale bars, 25 μm. **c**, Jitter plot quantifying O^6^-EG positivity with time following ENU injection. *n* = 3 mice per timepoint; 7 intestinal regions scored per mouse. **d**, Kaplan–Meier curves for *Apc*^*het*^ + ENU, *Apc*^*het*^, wild-type (WT) + ENU and wild-type mice aged until the humane endpoint. Mantel–Cox *P* value < 0.0001. *n* = 49 mice for *Apc*^*het*^ + ENU, 10 for *Apc*^*het*^, 32 for wild type + ENU and 5 for wild type. **e**, Number of intestinal tumours per mouse under indicated conditions. *n* = 5 mice for *Apc*^*het*^ + ENU, 8 for *Apc*^*het*^, and 20 for wild type + ENU. Two-sided Wilcoxon rank-sum tests. **f**, Representative wholemounts for *Apc*^*het*^ and *Apc*^*het*^ + ENU. S1–S5, small intestine; C1, proximal colon; C2, distal colon. Scale bars, 10 mm. **g**,**h**, Regional differences in tumour burden. *n* = 8 mice for *Apc*^*het*^ (**g**), 5 for *Apc*^*het*^ + ENU (**h**). **i**,**j**, Representative confocal micrographs showing an uncoloured tumour (**i**) and three examples of homotypic tumours (**j**). Scale bars, 200 μm. **k**, Frequency of Confetti labels in homotypic tumours. Counts based on 1,352 intestinal tumours. *n* = 5 mice. **l**, Confocal micrographs showing three examples of heterotypic tumours. **m**, Mean heterotypic fraction and regional distribution. *n* = 5 mice. Prox., proximal; SI, small intestine. Scale bars, 500 μm. In all box plots, the centre line shows the median, the bottom hinge shows the 25% quantile, the top hinge shows the 75% quantile, the bottom whisker shows the smallest observation greater than or equal to bottom hinge minus 1.5 × interquartile range (IQR), and the top whisker shows the largest observation less than or equal to the top hinge plus 1.5 × IQR. Source Data

**Extended Data Fig. 1:**
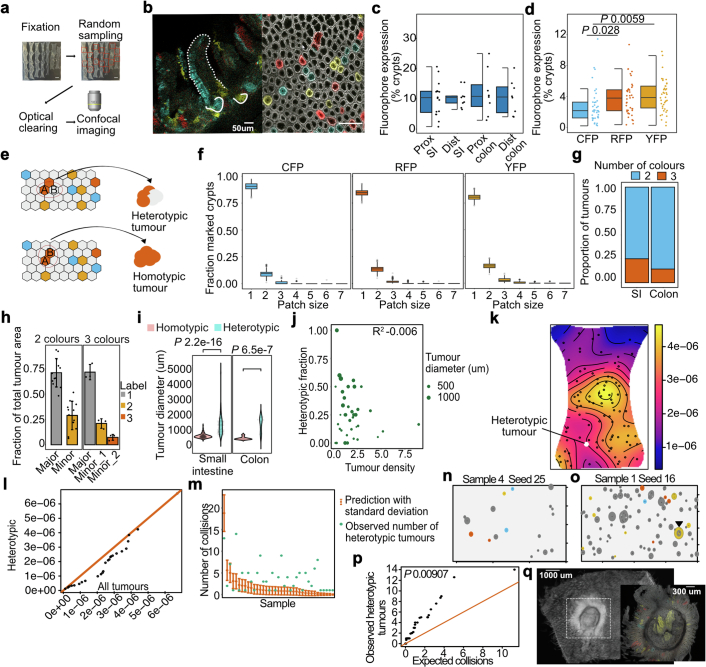
Confetti expression quantification and estimation of contribution of random collisions. **a**, Schematic illustrating tissue preparation steps prior to confocal imaging. **b**, Confocal micrograph of Confetti-labelled crypts in sagittal (left) and cross (right) sections. Tissue was collected from an *Apc*^*het*^ animal 10 days after tamoxifen induction. **c**, Box plot showing the frequency of Confetti fluorophore expression in different intestinal regions assessed. n = 41 intestinal segments from 7 mice. **d**, Box plot of frequency of individual Confetti fluorophores. Two-tailed t-test. **e**, Schematic illustrating the influence of patch size on the heterotypic fraction assessment. If an adenoma arises from two contiguous crypts with two different Confetti labels, the resulting tumour is identified as heterotypic. However, if it arises from a patch of similarly coloured crypts, it is identified as homotypic despite being polyclonal in origin. **f**, Box plot showing the simulation results for estimating patch sizes for individual fluorophores. 1000 simulations were performed for each fluorophore. **g**, Bar chart showing the number of Confetti colours in heterotypic tumours. **h**, Bar chart depicting the relative sizes of Confetti-labelled clones in heterotypic tumours. n = 13 tumours from 5 mice. **i**, Violin plot comparing the diameters of homotypic and heterotypic tumours in the small and large intestines. Based on 162 homotypic and 98 heterotypic tumours. Two-tailed t-test. **j**, Scatter plot of the heterotypic fraction against tumour density (number of tumours per 1000 crypts). Linear regression adjusted R^2^ −0.006. **k**, Representative heat map of the spatial tumour density for one intestinal segment. Tumours are marked by black dots, with the only heterotypic tumour in this segment labelled in white. **l**, Q-Q plot of heterotypic tumour spatial density against spatial density of all other tumours. Heterotypic tumours are not associated with a higher spatial density. **m**, Scatter plot showing the results of a Poisson model predicting the number of random tumour collisions for individual gut segments. The mean predicted number of collisions with the associated standard deviation is shown in red, with the green dot representing the observed number of heterotypic tumours for the segment. n = 37 samples from 10 mice. **n,o**, Examples of intestinal segments reconstructed following simulation of tumour initiation and growth. Random collision in **o** highlighted with black arrowhead. **p**, Q-Q plot of mean observed number of heterotypic tumours per imaged intestinal segment versus expected number of collisions in that segment based on simulation under conditions of random collision. Paired two-tailed t-test. **q**, Confocal micrograph of a segment of small intestine from a wildtype + ENU animal containing only one tumour. The inset shows that this tumour was heterotypic, consisting of at least two clones (uncoloured and yellow). Error bars denote s.d. (**h, m**). Details on the boxplots are provided in the Methods.

**Extended Data Fig. 2:**
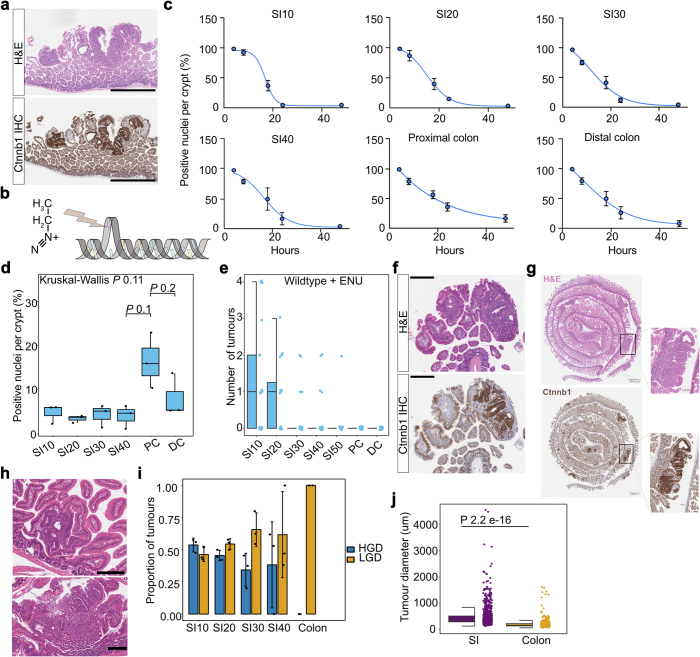
Characterisation of ethyl adduct clearance dynamics and ENU-mediated tumorigenesis. **a**, H&E and IHC staining for β-catenin in a longitudinal section from a small intestinal tumour in the *Apc*^*het*^ model in the absence of ENU. Scale bars 1000 μm. **b**, Schematic representation of ENU-mediated nucleotide alkylation. **c**, Decay curves for O-6-ethyl-guanine positivity following ENU administration. n = 3 animals per time point in each region. Error bars denote s.d. **d**, Box plot showing comparison of O-6-ethyl-guanine positivity at 48 h in the different intestinal regions. n = 25 half-crypts per region per animal. 3 animals used. Kruskal-Wallis test. **e**, Box plot showing the intestinal tumour burden by region in wildtype mice after ENU. n = 20 mice. **f**, H&E and β-catenin IHC in a small intestinal tumour in a wildtype + ENU mouse. Scale bars 300 μm. **g**, H&E- and β-catenin-stained swissroll sections from *Apc*^*het*^ + ENU model. **h**, Representative H&E-stained sections showing low-grade (top) and high-grade dysplasia (bottom) in tumours in *Apc*^*het*^ + ENU. **i**, Bar plot showing the distribution of tumours with low- and high-grade dysplasia across different regions in the *Apc*^*het*^ + ENU model. n = 30 tumours across 3 mice. Error bars represent standard deviation around mean. **j**, Box plot showing distribution of tumour sizes between small intestine and colon. Based on 868 small intestinal and 585 colonic tumors in 5 mice. Two-tailed t-test. Scale bars: 25 μm (h), 300 um (f), 1000 um (a,g). Details on the boxplots are provided in the Methods.

A tumour model based on *Villin-cre*^*ER*^*;Apc*^*fl/+*^;*Rosa*^*fl/Confetti*^ (hereafter *Apc*^*het*^*;Confetti*) mice and *N*-ethyl-*N*-nitrosourea (ENU) mutagenesis was adopted, that combined tissue-wide monoallelic loss of *Apc* with sporadic activation of the *Confetti* reporter (Fig. 1a). The small patch sizes of *Confetti-*marked crypts maximize the mosaicism that facilitates identification of polyclonality compared with that used in previous approaches, which depended on somatic mosaics^7,17,20,22^. Around 10% of crypts expressed a *Confetti* colour (Extended Data Fig. 1a–f). Subsequent ENU treatment initiated tumours with high multiplicity along the intestinal tract following rapidly resolved DNA damage (Fig. 1b–h and Extended Data Fig. 2). Tumours displayed increased levels of nuclear β-catenin, consistent with sporadic loss of the second *Apc* allele (Extended Data Fig. 2f,g). We assessed the clonal status of tumours on the basis of heterotypia in *Confetti* expression in these ‘*Apc*^*het*^ plus ENU’ mice. Most tumours were uncoloured and of initially unknown clonal status (Fig. 1k). Of tumours that expressed *Confetti*, 60% were homotypic and 40% were heterotypic, and thus potentially monoclonal and polyclonal in origin, respectively (Fig. 1i–m and Extended Data Fig. 1g–i).

To eliminate collisions as an explanation for heterotypia due to high tumour incidence, we first confirmed that there was no obvious relationship between tumour density on average across gut segments analysed and the heterotypic fraction (linear regression adjusted *R*^2^ = −0.006) (Extended Data Fig. 1j). Next, a higher-resolution analysis taking account of clustering ‘hotspots’ within individual segments of bowel revealed that heterotypic tumours were not enriched in regions of higher tumour density (Extended Data Fig. 1k,l). To further take account of tumour size as well as density, the number of expected collisions was predicted assuming a Poisson distribution, to show that more heterotypic tumours are observed than expected by random collision theory (Extended Data Fig. 1m). The additional effect of tumour growth on the probability of collisions was tested by simulations and also could not explain the observed incidence of heterotypic tumours (Extended Data Fig. 1n–p). Of note, heterotypia was also observed in the small number of tumours found in either *Apc*^*het*^ control mice not receiving ENU or in wild-type mice receiving ENU (Extended Data Fig. 1q).

## *Apc* mutations define polyclonal tumours

**Fig. 2:**
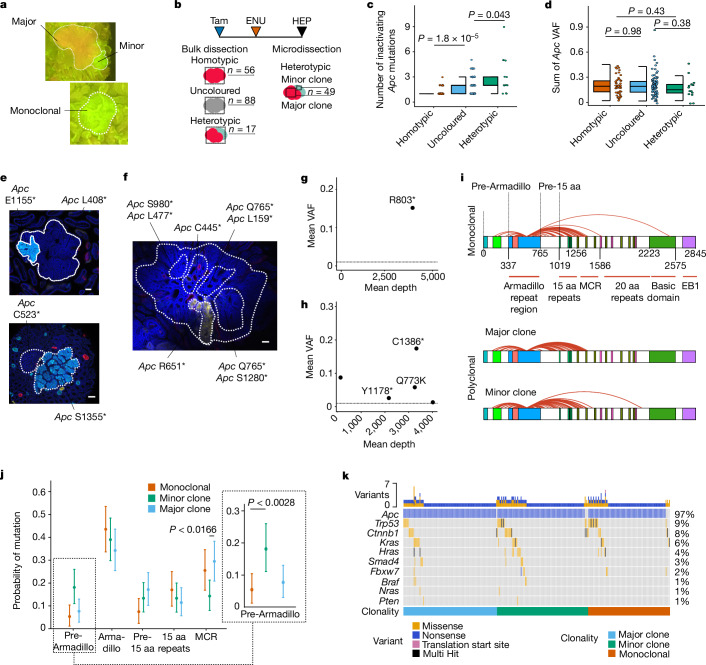
Polyclonal and monoclonal tumours are distinguished by *Apc* mutational profiling. **a**, Fluorescence dissecting microscope view of a heterotypic tumour (top) and a homotypic tumour (bottom). **b**, Schematic of experimental approach. Tumours were either bulk- or micro-dissected before targeted amplicon sequencing. HEP, humane endpoint. **c**, Number of inactivating (nonsense-only) mutations in *Apc* for each bulk-sequenced tumour. Based on 56 homotypic, 88 uncoloured and 17 heterotypic tumours from 10 *Apc*^het^ + ENU mice. Two-sided Wilcoxon rank-sum tests. **d**, Sum of the VAFs of the inactivating *Apc* mutations for each tumour in the indicated bulk-dissected groups. *n* = 148 tumours. Two-sided Wilcoxon rank-sum tests. **e**,**f**, Confocal images of micro-dissected heterotypic tumours overlaid with detected high-impact *Apc* variants (**e**) and a large micro-dissected heterotypic tumour (**f**). Scale bars, 100 μm. **g**,**h**, Representative clonality plots showing mean VAF versus mean sequencing depth for variants shown for a homotypic (**g**) and an uncoloured (**h**) tumour. Dotted line represents minimum VAF threshold for variant calls. **i**, Arch diagram overlaid on a schematic of the APC protein to compare high-impact *Apc* mutations in monoclonal and polyclonal tumours. Arches begins at codon 580, representing the Cre-mediated recombination event of the transgenic *Apc* allele. *n* = 94 monoclonal tumours, 105 major and 105 minor clones. EB1, EB1-binding region; MCR, mutation cluster region; aa, amino acid. **j**, Non-parametric bootstrap analysis showing the probability of mutation in each of the pre-defined *Apc* bins for monoclonal tumours, and major and minor clones. Data are mean ± 95% confidence interval. *n* = 94 samples per group. Inset, magnified view of the Pre-Armadillo bin, highlighting the significant difference between monoclonal tumours and minor clones. **k**, Oncoprint of mutational patterns among the indicated groups. Percentages on the right denote fraction of samples with detected mutations in particular gene. Source Data

**Extended Data Fig. 3:**
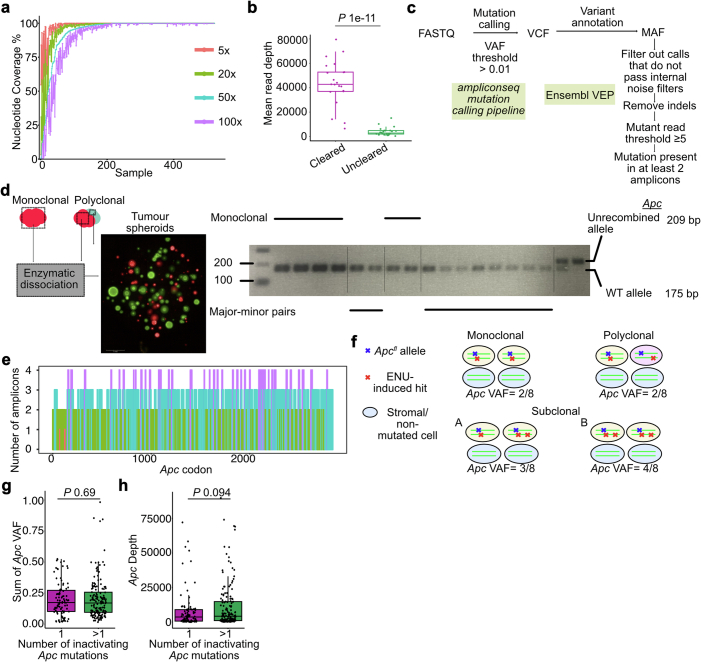
Targeted amplicon sequencing of *Apc.* **a**, Plot showing the overall sequencing coverage across all samples at various depths. n = 522 samples. **b**, Box plot showing the mean read depth between optically cleared and uncleared samples. n = 21 cleared tumours and 24 uncleared tumours. **c**, Schematic showing the pre-processing and mutation-calling pipeline. **d**, Schematic showing derivation of tumour spheroids used to confirm recombination of the transgenic *Apc* allele in both heterotypic and homotypic tumours. PCR for the floxed allele of *Apc* was performed on DNA derived from spheroids. Electrophoregram showing complete loss of the unrecombined band (209 bp) in monoclonal tumours and major-minor pairs. Positive controls shown on the right of the gel are from liver tissue of an *Apc*^*het*^ mice (two bands) and *Apc*^*fl/fl*^ animal without tamoxifen (one band). n = 6 monoclonal tumour spheroids and 10 major-minor pairs from 5 polyclonal tumours. **e**, Plot showing the number of amplicons covering each amino acid position in *Apc*. **f**, Schematic showing the influence of the clonal/subclonal structure on the resulting *Apc* VAF. Assuming a constant tumour fraction of 0.5, the sum of *Apc* VAFs is higher in situations of branching evolution (with more than one *Apc* mutation per tumour cell). **g, h**, Boxplot of *Apc* driver VAF (**g**) and *Apc* sequencing depth (**h**) for monoclonal tumours (one *Apc* mutation) and polyclonal tumours (two or more mutations). n = 154 monoclonal and 93 polyclonal tumours. Two-tailed Wilcoxon test in **b**, **g** and **h**. Details on the boxplots are provided in the Methods.

A ten-gene targeted amplicon panel that included *Apc* was created for bulk sequencing of excised tumours with different *Confetti* outcomes to identify drivers of tumour formation (Fig. 2a,b. Extended Data Fig. 3 and Supplementary Table 1). Inactivating mutations of *Apc* were identified in homotypic, uncoloured and heterotypic tumours (161 out of 183 tumours contained *Apc* mutations). Most (82%) homotypic tumours contained only a single *Apc*-inactivating nonsense mutation. By contrast, around 20% of heterotypic tumours had a single *Apc* mutation, with the remaining containing between 2 and 9 *Apc* mutations (Fig. 2c). As the model only requires a single *Apc* mutation to complement the tissue-wide Cre-mediated loss of the first allele, the *Apc* variant allele fraction (VAF) directly reflects the tumour fraction in each sample sequenced (Extended Data Fig. 3d). Notably, the distribution of calculated tumour fraction values for all three tumour categories based on Confetti classification were indistinguishable when the VAF values of all *Apc* mutations in each tumour were summed (Fig. 2d). This excludes that multiple *Apc* mutations in heterotypic tumours might arise from a branching model of tumour evolution whereby subclones acquire subsequent mutations (Extended Data Fig. 3f). Notably, there was no difference in the sum of the *Apc* VAFs and sequencing read depth between samples with one or more *Apc* mutations, indicating that the number of *Apc* mutations detected cannot be attributed to differences in tumour purity or sequencing efficiency (Extended Data Fig. 3g,h).

**Extended Data Fig. 4:**
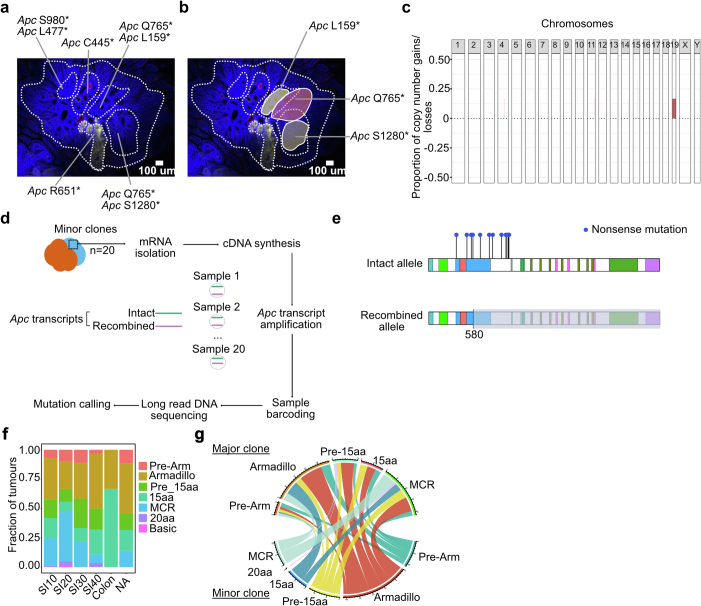
Explaining the multiple *Apc* hits. **a**, Microdissection of heterotypic tumour with detected *Apc* nonsense mutations overlaid. **b**, Possible organisation of clones that would explain sequencing results obtained in **a**. **c**, Copy number analysis summary showing relative quiescence of copy number alterations in *Apc*^*het*^ + ENU tumours. n = 6 tumours. **d**, Schematic showing experimental design for mutation phasing experiment. **e**, Lollipop plot showing *Apc* mutation location and allelotypes from microdissected minor clones. n = 11 minor clones with detected *Apc* mutations. **f**, Stacked bar chart showing the fraction of tumours across different intestinal regions with mutations in particular *Apc* domains. n = 59 tumours in SI10, 51 in SI20, 25 in SI30, 18 in SI40, 4 in colon, and 99 tumours with no recorded location information. **g**, Circos plot showing combinations of *Apc* domain mutations between the major and minor clone pairs within polyclonal tumours.

In a more detailed analysis, different coloured regions from 49 heterotypic tumours were dissected from wholemount preparations and sequenced. This confirmed that most larger (major) and smaller (minor) clones contained exclusive *Apc*-inactivating mutations, commonly only one and occasionally two (Fig. 2e,f and Extended Data Fig. 4a,b).

The status of uncoloured tumours was then assessed on the basis of bulk sequencing and an assignment of clonality was made for each of 88 such tumours on the basis of the number of *Apc* mutations. Samples containing a single somatic *Apc* mutation were classed as monoclonal. For polyclonal tumours, divergent VAF values for *Apc* mutations commonly enabled assignment of major and minor clones that contributed to tumour mass (Fig. 2g,h). This approach was validated owing to the sparsity of copy number alterations induced by ENU in this model (Extended Data Fig. 4c). Comparing the relative frequencies of mutation in different functional domains of *Apc* for the different clonal tumour components demonstrated that early truncating mutations landing N-terminal to the Armadillo repeat region were under-represented in monoclonal tumours compared with the minor clones of polyclonal tumours (Fig. 2i,j). Long-read sequencing performed on a subset of minor clones revealed that the detected *Apc* mutations arise on the non-recombined allele, confirming that these clones also depend on biallelic *Apc* inactivation (Extended Data Fig. 4d,e). No regional selection for particular *Apc* mutations was observed and similarly, within-tumour paired analysis of major and minor clone mutated domains revealed no selected combinations (Extended Data Fig. 4f,g). Mutations in the nine other genes in the panel including *Trp53*, *Ctnnb1* and *Kras* were found but were not differentially enriched within tumour clones (Fig. 2k).

## Clonal RAS–MYC reciprocity

**Fig. 3:**
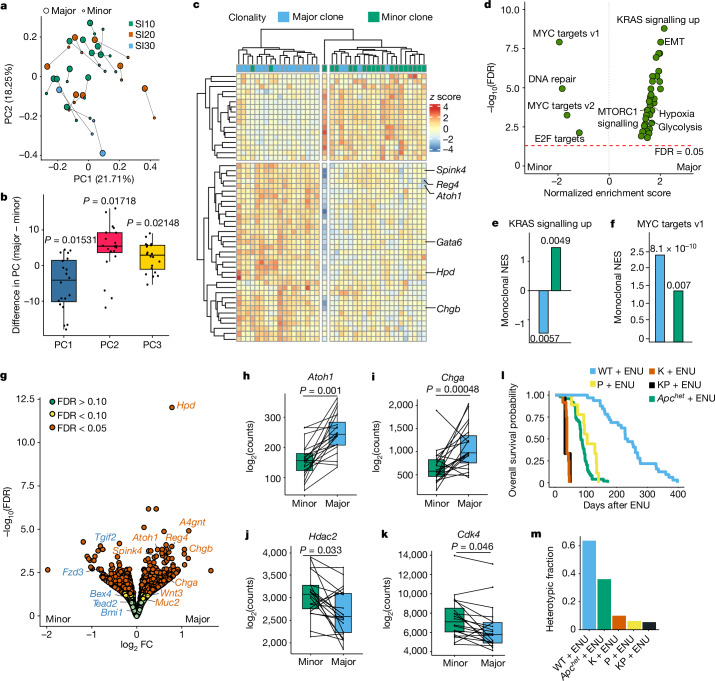
Transcriptional profiling identifies clonal cooperation by reciprocity in RAS and MYC pathways. **a**, PCA of comparison between major and minor clones, showing the first two principal components (PC1 and PC2). Major clones connect to corresponding minor clones. Colour represents location in the small intestine. **b**, Box plot of difference in principal component within major–minor pairs for each of PC1–PC3. Two-tailed one-sample Wilcoxon signed-rank test. **c**, Heat map with hierarchical clustering shows top 50 differentially expressed genes between the major and minor clones. **d**, Volcano plot showing normalized gene set enrichment scores for Hallmark Pathways in the comparison between major and minor clones. Dotted line denotes a false discovery rate (FDR) of 0.05. EMT, epithelial–mesenchymal transition. **e**,**f**, NES of Kras_Signaling_Up (**e**) and Myc_Targets_v1 (**f**) for Hallmark Pathways in monoclonal tumours relative to major clones and minor clones. *n* = 20 biological replicates (20 monoclonal tumours from 2 mice). Values represent FDR from gene set enrichment analysis. **g**, Volcano plot of differentially expressed genes between major and minor clones. Secretory genes are labelled in red and stem cell genes are labelled in blue. FC, fold change. **h**–**k**, Transcript counts for *Atoh1* (**h**), *Chga* (**i**), *Hdac2* (**j**) and *Cdk4* (**k**) in individual major and minor pairs. Paired two-tailed Wilcoxon tests. **l**, Kaplan–Meier survival curves for wild type + ENU, *Trp53*^*null*^ (P) + ENU, *Kras*^*G12D/+*^ (K) + ENU, *Kras*^*G12D/+*^*;Trp53*^*null*^ (KP) + ENU and *Apc*^*het*^ + ENU. *n* = 32 mice for wild type + ENU, 9 mice for P + ENU, 12 mice for K + ENU, 5 mice for KP + ENU and 49 mice for *Apc*^*het*^ + ENU. **m**, Heterotypic fraction across models described in **l**. Assessment based on 22 coloured tumours for wild type + ENU, 249 for *Apc*^*het*^ + ENU, 90 for *Kras*^*G12D/+*^ + ENU, 144 for *Trp53*^*null*^ + ENU and 185 for *Kras*^*G12D/+*^*;Trp53*^*null*^ + ENU. *n* = 20 biological replicates per group in **a**–**k**. Source Data

**Extended Data Fig. 5:**
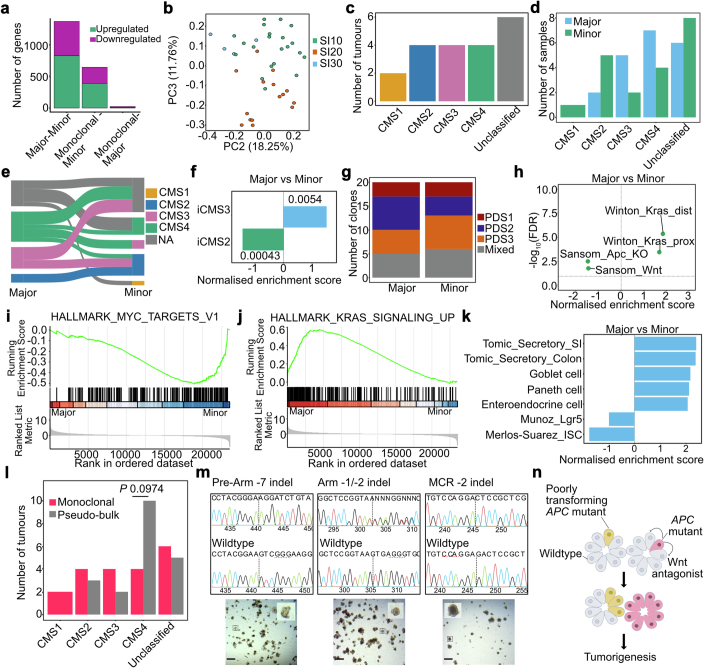
Clonal architecture influences transcriptional heterogeneity. **a**, Bar chart comparing the number of differentially expressed genes between major and minor clones, monoclonal versus minor clones, and monoclonal versus major clones at an adjusted p-value < 0.05. **b**, Principal component (PC) analysis of major-minor clones showing relationship between PC2 and PC3. **c,d**, Bar chart of consensus molecular subtype (CMS) prediction for 20 monoclonal tumours (**c**) and, 20 major and minor clones (**d**) based on the MmCMS algorithm (option C). **e**, Sankey diagram showing pairing of major-minor clones according to their CMS status. **f**, Bar chart showing results of gene set enrichment analysis between major and minor clones using iCMS signatures. Values denote FDR from GSEA. **g**, Stacked bar chart showing results of pathway-derived subtyping (PDS) for major and minor clones. **h**, Volcano plot showing gene set enrichment scores for major-minor clone comparison using mouse-specific Wnt and Kras signalling signatures. **i**, Gene set enrichment scores for intestinal cell-type-specific signatures. **j,k**, Enrichment plots for Hallmark_Myc_Targets_v1 (**j**) and Hallmark_Kras_Signaling_Up (**k**) gene sets for major-minor clone comparison. **l**, Bar chart of CMS classification of 20 monoclonal tumours and 20 reconstructed polyclonal tumours (pseudo-bulk). Two-tailed z-test of equality of proportions. **m**, Sanger sequencing of clonal Pre-Armadillo (S96*), Armadillo (T619*) and MCR (F1378*) *Apc* knockouts with representative brightfield images showing the respective organoids. Scale bars: 50 μm. **n**, Schematic showing suggested mechanism through which different level of Wnt antagonism can contribute to polyclonal tumorigenesis. A poorly-transforming *APC* mutant intestinal stem cell (ISC) is unable to outcompete neighbouring wildtype ISCs due to inadequate Wnt antagonist secretion, unless in the presence of another *APC* mutant with supercompetitor behaviour.

To identify phenotypic differences in the clones comprising polyclonal tumours, major and minor clones from heterotypic tumours were again dissected and analysed by transcriptional profiling (Fig. 3 and Extended Data Fig. 5). The pairing of clones within tumours uniquely controls for mouse or regional gut difference and analysis retained this integral relationship. Principal components analysis (PCA) revealed a consistent separation (17 out of 20 tumours) of major and minor clones along PC2, where PC1 and PC3 separated on mouse and intestinal location differences, respectively (Fig. 3a,b and Extended Data Fig. 5b). Hierarchical clustering consistently segregated major and minor clones, indicating that these tended to similarity within that classification rather than in pairs common to the same tumour (Fig. 3c). CRC has been classified into four consensus molecular subtypes (CMS) based on bulk RNA expression signatures, and this classification has been shown to have prognostic value^23,24^. CMS1 correlates with hypermutated microsatellite-unstable cancers with deficient mismatch repair. By contrast, the remaining subtypes are typically microsatellite-stable but can be chromosomally unstable: CMS2 (canonical with high levels of WNT and MYC activation), CMS3 (metabolic with enrichment for *KRAS* mutations), and CMS4 (mesenchymal with epithelial–mesenchymal transition and stromal features). About 10% of tumours contain a mixture of these signatures and remain unclassified by existing algorithms. Tumour classification based on CMS revealed that 40% of minor clones remained unclassified, although this was not a statistically significant enrichment (Extended Data Fig. 5c,d). Their within-tumour pairing indicated that these unclassified minor clones predominantly associated with CMS3 and CMS4 major clones (Extended Data Fig. 5e). Applying the more refined intrinsic CMS (iCMS) classification that focuses on epithelial properties showed relative enrichment for iCMS3 (active RAS signature) in major clones and iCMS2 (increased expression of stem cell and MYC signatures) in minor clones (Extended Data Fig. 5f). Enrichment analysis for Hallmark Pathways indicated that major clones had increased KRAS signalling (normalized enrichment score (NES) = 2.09, *q*-value = 1.58 × 10^−9^) and depleted for MYC signalling relative to minor clones (NES = −1.99, *q*-value = 1.32 × 10^−8^) (Fig. 3d and Extended Data Fig. 5h–j). KRAS signalling in monoclonal tumours showed a higher net enrichment score than found in minor clones but a lower one than for major clones. By contrast, monoclonal tumours were enriched in expression of targets of MYC compared to both major and minor clones (Fig. 3e,f). Analysis of differentially expressed genes identified that major and minor clones were also enriched for expression of secretory and stem cell or replicative genes, respectively (Fig. 3g–k and Extended Data Fig. 5k).

These findings suggest that *Apc* loss-of-function mutations have the capability to initiate polyclonal tumour development through interactions between clonal populations that have sub-optimal activation of pathways associated with oncogenic transformation including that of *Kras*. To assess whether this capability is dependent on an imbalance in pathway activation between founding cells, the *Kras*^*LSL-G12D*^ allele was intercrossed to *Villin-cre*^*ER*^*;Rosa*^*fl/Confetti*^ mice and treated with tamoxifen and subsequently ENU. Analysis of tumours in these *Kras*^*G12D/+*^*;Confetti* mice confirmed that 90% of tumours were likely to be monoclonal, as indicated by homotypia for Confetti (Fig. 3l,m). To minimize loss of ENU-damaged cells and thus maximize clonal availability, an additional *Trp53*^*fl/fl*^ allele was introduced and the experiment was repeated in *Trp53*^*null*^*;Confetti* mice with and without the *Kras*^*G12D*^ allele. Again, this confirmed the resultant tumours to be overwhelmingly homotypic for Confetti and therefore have a high probability being monoclonal (Fig. 3l,m). By contrast, around 60% of 22 coloured (and therefore informative) tumours arising at low multiplicity and long latency in wild-type mice following ENU were heterotypic for Confetti, indicating that polyclonal tumorigenesis is not dependent on *Apc* field effects (Fig. 3m).

**Fig. 4:**
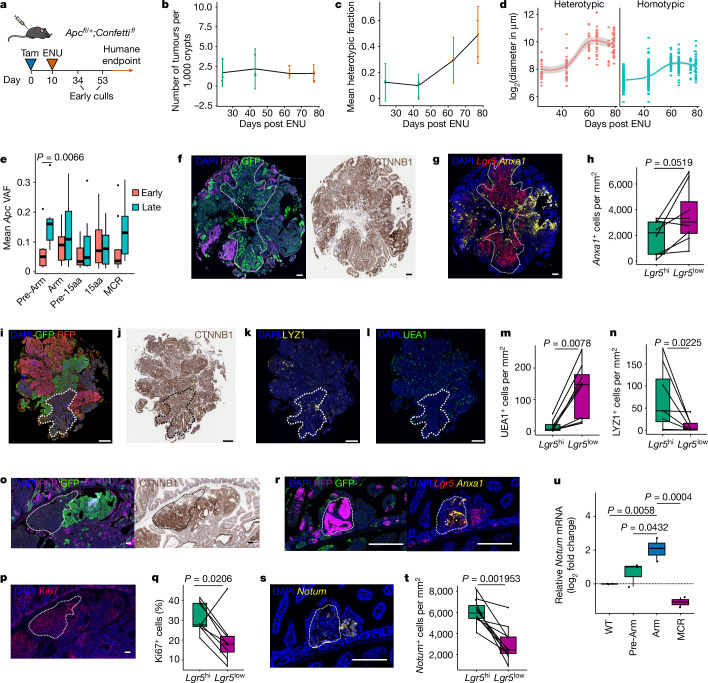
Growth dynamics and clonal phenotyping of heterotypic tumours. **a**, Schematic of experimental approach. Mice were collected at early timepoints or aged until humane endpoint. **b**,**c**, Normalized number of tumours (**b**) and mean heterotypic fraction (**c**) after ENU. Early culls, green; humane endpoint, red. *n* = 3 mice, except at 63 days, where *n* = 2. Data are mean ± s.d. **d**, Growth curves for heterotypic and homotypic tumours. *n* = 3 mice at 24 and 43 days, 1 for other timepoints. Mixed-effects model for exponential growth phase, two-tailed *t*-test *P* < 0.0001. **e**, *Apc* VAFs for indicated domains. Tumours are classed as early (less than 80 days after ENU) and late (more than 80 days after ENU). *n* = 27 samples in the early group and 34 samples in the late group. Two-tailed *t*-test with Benjamini–Hochberg correction. **f,g**, Immunofluorescence staining for GFP and RFP, with β-catenin immunohistochemistry (**f**) or duplex RNAscope staining for *Lgr5* and *Anxa1* (**g**). **h**, *Anxa1* positivity in *Lgr5*^hi^ and *Lgr5*^low^ clones within heterotypic tumours. **i**–**l**, Serial sections of heterotypic tumour. GFP and RFP immunofluorescence (**i**), β-catenin immunohistochemistry (**j**), lysozyme-1 (LYZ1) immunofluorescence (**k**) and UEA1 immunofluorescence (**l**). **m**,**n**, Quantification of UEA1 (**m**), LYZ1 (**n**) in *Lgr5*^hi^ and *Lgr5*^low^ clones within heterotypic tumours. **o**,**p**, Serial sections with immunofluorescence for GFP and RFP and immunohistochemistry for β-catenin (**o**) and immunofluorescence for Ki67 (**p**). **q**, Quantification of Ki67 positivity in *Lgr5*^hi^ and *Lgr5*^low^ clones within heterotypic tumours. **r**,**s**, Serial sections with immunofluorescence staining for GFP and RFP and duplex RNAscope staining for *Lgr5* and *Anxa1* (**r**) or fluorescent RNAscope staining for *Notum* (**s**). **t**, *Notum* positivity in *Lgr5*^hi^ and *Lgr5*^low^ clones within heterotypic tumours. *n* = 12 from 3 mice. **u**, *Notum* expression in *Apc*-mutant organoids. Three independent experiments per sample. Tukey’s multiple comparisons test. In **h**,**m**,**n**,**q**, *n* = 8 tumours from 3 mice. Paired two-tailed Wilcoxon test. Scale bars: 100 μm (**f**,**g**,**i**–**l**,**o**,**p**), 50 μm (**r**,**s**). Source Data

We next investigated whether the clonal status of tumours changes with time (Fig. 4a). Microscopic analysis established that although the heterotypic fraction increased with time, the number of tumours did not (Fig. 4b,c). This implicates tumour growth in the development of polyclonality and supports clonal recruitment as the underlying mechanism^18^. In humans, polyp size is recognized as one of the most important risk factors that determines the risk of developing cancer^25,26^. To determine whether polyclonality could act to increase the risk of cancer development and progression, we investigated the growth characteristics of heterotypic and homotypic tumours. Changing tumour size distributions with time established that heterotypic tumours grew to a larger overall size with a fourfold faster rate of exponential growth than homotypic tumours, confirming observations in other mouse models^7,17^ (Fig. 4d). To determine whether the accelerated growth rate of heterotypic tumours was related to the underlying *Apc* mutational profile, tumours were stratified into those recovered early or late (before or after 80 days, respectively). This indicated a significant under-representation of N-terminal truncations in the early-recovered tumours that provides some evidence for their recruitment as tumours grew (Fig. 4e). To link these observations to tumour phenotype, the transcriptome of heterotypic tumours was reconstructed by sampling transcripts from constituent major and minor clones. This revealed an enrichment of these pseudo-bulk polyclonal tumours in CMS4, which is classically associated with a more aggressive phenotype^27^ (Extended Data Fig. 5l).

## Clonal properties confer heterogeneity

It has recently been proposed that human and mouse intestinal tumours develop from a fitness landscape that includes both canonical homeostatic LGR5^hi^ stem cells and regenerative LGR5^low^ stem cells defined by fetal marker expression such as *Anxa1*^28,29^. Tumours retain populations of both stem cell types reflecting a more plastic state^30^. To explore whether there were differences between clonal constituents in their representation of different stem cell types a tissue microarray of heterotypic tumours was probed for expression of stem cell (*Lgr5* and *Anxa1*), proliferative (Ki67) and secretory (UEA1 and LYZ1) markers (Fig. 4). This revealed segregation of mutually exclusive *Lgr5*^hi^ and *Anxa1*^hi^ tumour regions that overlaid the clonal territories defined by Confetti (Fig. 4f–h). Paneth cell markers are known to be increased with *Apc* loss and to associate with *Lgr5* expression^31–33^. Comparing goblet and Paneth cells markers (UEA1 and LYZ1, respectively) identified a reciprocal relationship with the former associating with *Lgr5*^low^ regions and the latter associating with *Lgr5*^hi^ regions (Fig. 4i–n). *Lgr5*^hi^ regions also contained more proliferating cells than *Lgr5*^low^ regions (Fig. 4o–q). Together, these results support that polyclonal tumours are maintained by different stem cell states that are spatially segregated and defined by their *Apc* mutational status.

In an attempt to reconcile our findings with the previously described supercompetitor behaviour of *Apc* mutants, we probed the expression of the Wnt antagonist NOTUM in serial sections from heterotypic tumours (Fig. 4r,s). This revealed a clear difference in the level of *Notum* RNA expression within tumours that overlaid Confetti-defined subclones, with *Lgr5*^hi^ clones expressing a higher level of *Notum* (Fig. 4t). To investigate whether this difference depends on the nature of the *Apc* mutation, we created an isogenic allelic series of *Apc* truncations in organoids to reflect the main driver events identified in the model by sequencing. This analysis confirmed that the relative expression of *Notum* was significantly lower in the variants N- or C-terminal to the Armadillo domain (Fig. 4u and Extended Data Fig. 5m). This indicates that *Apc*-mutant clones are likely to differ in their ability to influence neighbouring cells and, from the observations on polyclonal tumours reported here, that supercompetitive behaviour also promotes clonal cooperation (Extended Data Fig. 5n).

Although often initiated by a ‘just right’ combination of *Apc* mutation that preserves at least one β-catenin binding domain in one allele, dysregulation of Wnt signalling is an ongoing process in the progression of CRCs^34^. Loss-of-function mutations in Wnt antagonists and modifiers continue to be selected^21,35–38^. The development of polyclonal tumours from clones with distinct *Apc* mutational profiles suggests that just right conditions for tumour initiation can be achieved by cooperation between founder clones reciprocating in their perturbation of APC–MYC and KRAS pathways.

Adenomas represent the earliest recognized stage of tumour formation that leads to cancer in patients, but few profiling studies have been performed. However, human analyses accompanying this study confirm that polyps arising from genetic predisposition or sporadically frequently have complex clonal origins^39^. The degree to which polyclonal polyps have an increased risk of developing into carcinomas remains uncertain but their larger size already accommodates one such risk factor^40^. The extent to which *APC* mutational profiling indicates an aetiology for sporadic human polyps similar to that described here will also be important for assigning risk of progression associated with the phenomenon. Finally, clonal cooperation may no longer be required in more advanced cancers^41^. Multiregional sampling of such cancers that reconstruct initial trunk mutations and later branching ones would support this view^42–47^. However, such analyses require identification of a founder clone and polyclonality may be under-reported for this reason^48,49^. Where this constraint has been addressed, polyclonal sporadic CRCs have been identified^50^.

## Methods

### Mice

The intestinal epithelium-specific inducible Cre (*Villin-creERT2)*^51^ (JAX020282) line was crossed with *Apc*^*fl/+*^ (ref. ^52^) and *R26R-Confetti*^53^ (JAX017492) lines on a C57BL/6 background to obtain mice heterozygous for these alleles. Additionally, the LSL-*Kras*^*G12D*^^54^ (JAX008179) and *Trp53*^*fl/fl*^^55^ alleles were used in some experiments. Genotyping was performed by Transnetyx using real-time PCR.

### Animal husbandry

Male and female mice of at least 8 weeks of age were used for the experiments. Mice were housed under controlled conditions (temperature (21 ± 2 °C), humidity (55 ± 10%), 12 h light/dark cycle) in individually ventilated cages in a specific pathogen-free facility (tested according to the recommendations for health monitoring by the Federation of European Laboratory Animal Science Associations). Food and water were provided ad libitum. None of the mice had been involved in any procedure prior to the study. For survival curve generation, the mice were aged until they showed pre-defined clinical signs of tumour burden (anaemia, hunching, and loss of body condition). No mice were allowed to exceed these pre-defined endpoints. No randomization or blinding was used. Sample sizes were determined from the results of preliminary experiments. All animal experiments were performed in accordance with the guidelines of the UK Home Office under the authority of a Home Office project licence (PD5F099BE) approved by the Animal Welfare and Ethical Review Body at the CRUK Cambridge Institute, University of Cambridge.

### Field induction and mutagenesis

Induction of tumour suppressor and/or oncogene fields (along with the Confetti multicolour lineage reporter) was triggered by a single intraperitoneal injection of 4 mg tamoxifen (Merck T5648) dissolved in ethanol/sunflower oil (1:9). Chemical mutagenesis was performed exactly 10 days after field induction using 200 mg kg^−1^ ENU dissolved in ethanol/phosphate-citrate buffer (1:9) given intraperitoneally.

### Tissue clearing

Mice were culled by cervical dislocation. The whole intestine was dissected, flushed with cold PBS, cut longitudinally, and wholemounted. Following fixation in 4% paraformaldehyde for 24 h at 4 °C, the tissue was washed in PBS and randomly chosen segments of the bowel were excised. Optical clearing was performed using the CUBIC protocol^56^. In brief, excised segments were incubated with CUBIC-1a solution (10% urea, 5% *N*,*N*,*N*′,*N*′-tetrakis(2-hydroxypropyl) ethyl-enediamine, 10% Triton X-100 and 25 mM NaCl in distilled water) at 37 °C for 7–10 days with alternate day solution changes. DAPI was used for nuclear counterstaining at a dilution of 1:1,000. The cleared tissue was then washed in PBS for 24 h. Additional clearing and refractive index matching were performed with Rapiclear 1.52 (SunJin Labs 152002) for 24 h. Finally, the samples were mounted in a 0.25 mm i-Spacer (Sunjin Labs) for confocal imaging.

### Microscopy

Images were acquired on a Leica SP5 TCS confocal microscope (LAS software v2.8.0, Leica) with a 10× objective, 1.4–1.7 optical zoom and 8–12 μm *z*-steps throughout the whole thickness of the tissue. Image analysis was performed using ImageJ software^57^. All identified tumours had their Confetti status manually assessed at all of the acquired *z* positions. A tumour was only identified as heterotypic if it showed evidence of glands of at least two Confetti colours or one Confetti colour in the presence of unlabelled glands. Single intermixed glands were disregarded for the purpose of determining heterotypic status, as they most probably represent entrapped normal crypts.

### Immunohistochemistry

Wholemounts or swiss rolls were fixed in 4% paraformaldehyde for 24 h at 4 °C before paraffin embedding and sectioning by the CRUK CI Histopathology Core. Haematoxylin and eosin (H&E) staining was performed using an automated ST5020 Multistainer (Leica Biosystems). Staining for β-catenin and *O*-6-ethyl-guanine was performed on Leica’s automated Bond-III platform in conjunction with the Polymer Refine Detection System (Leica, DS9800). In brief, epitope retrieval was performed using Leica Epitope Retrieval Solution 1 (Leica, AR9961) at 100 °C. Blocking was performed with Protein Block Buffer (Dako, X090930-2). Following incubation with primary antibody against β-catenin (0.25 μg ml^−1^, mouse, 610154, BD Biosciences) or O-6-ethyl-guanine (0.5 μg ml^−1^, rat, SQX-SQM001, Squarix Biotechnology), sections were incubated with secondary antibody (rabbit anti-rat, Bethyl Laboratories, A110-322A at 1:250 or rabbit anti-mouse IgG1, Abcam, ab125913 at 1:1,500) before development and mounting. For β-catenin staining, an additional mouse-on-mouse blocking step was performed.

### Immunofluorescence on paraffin sections

Heat-mediated epitope retrieval was performed on rehydrated 3-μm-thick paraffin sections in 10 mM sodium citrate (pH 6.0). The sections were then incubated in blocking solution (10% donkey serum and 0.05% Tween-20 in PBS) at room temperature for 30 min. Primary antibodies against RFP (1:100, rabbit, R10367, Thermo Fisher), GFP (1:100, chicken, ab13970, Abcam), Ki67 (1:100, rat, 14-5698-82, Thermo Fisher), or lysozyme (1:100, goat, sc-27958, Santa Cruz) were diluted in blocking solution, in which sections were then incubated in the dark at 4 °C for 12 h. Sections were washed and incubated with fluorophore-conjugated secondary antibodies (donkey anti-rabbit A31572, goat anti-chicken A11039, donkey anti-goat A21447, Thermo Fisher) diluted 1:200 in 0.05% Tween-20 in PBS for 45 min at room temperature. DAPI (1:1,000) was used for nuclear counterstaining along with native Ulex Europaeus Lectin 1 (UEA1) (AbD Serotec, 9420-00024) used at 1:200. After washing, the stained sections were mounted using ProLong Gold Antifade Mountant (Thermo Fisher, P36930).

### RNAscope

Simultaneous detection of *Lgr5* and *Anxa1* and detection of *Notum* were performed on paraffin embedded sections using Advanced Cell Diagnostics (ACD) RNAscope 2.5 LS Duplex Reagent Kit (322440), RNAscope 2.5 LS Probe- Mm- Anxa1 (509298), RNAscope 2.5 LS Probe-Mm-Lgr5-C2 (312178-C2), and RNAscope 2.5 LS Probe-Mm-Notum-C1 (428988-C1) (ACD). Three-micrometre-thick sections were baked for 1 h at 60 °C before loading onto a Bond RX instrument (Leica Biosystems). Slides were deparaffinized and rehydrated on board before pre-treatments using Epitope Retrieval Solution 2 (AR9640, Leica Biosystems) at 95 °C for 15 min, and ACD Enzyme from the Duplex Reagent kit at 40 °C for 15 min. Probe hybridization and signal amplification were performed according to manufacturer’s instructions. Fast red detection of C2 was performed on the Bond Rx using the Bond Polymer Refine Red Detection Kit (Leica Biosystems, DS9390) according to ACD protocol. Slides were then removed from the Bond Rx and detection of the C1 signal was performed using the RNAscope 2.5 LS Green Accessory Pack (ACD, 322550) according to kit instructions. Slides were heated at 60 °C for 1 h, dipped in Xylene and mounted using VectaMount Permanent Mounting Medium (Vector Laboratories, H-5000). The slides were imaged on the Aperio AT2 (Leica Biosystems) to create whole slide images. Images were captured at 40× magnification, with a resolution of 0.25 μm per pixel.

### Immunostaining quantification

All histological quantification was performed using QuPath (v.0.4.3; https://github.com/qupath/qupath)^58^. Annotations based on *Confetti* status were first manually created for each heterotypic tumour using a section stained for RFP and GFP. Positive cells for other markers were then identified using the positive cell detection feature with intensity threshold of 5 and a nucleus background radius of 8 μm, using DAPI as nuclear marker. For chromogenic or fluorescent duplex RNAscope staining, immunofluorescent detection of LYZ1 and UEA1, results were reported as the number of positive cells per unit area of the annotation. For Ki67 staining, results were reported as percentage of DAPI-positive cells.

### Tumour microdissection

Confocally imaged intestinal segments were washed with PBS and pinned onto black silicone pads. Tumours were visualized under a fluorescence dissecting microscope (Leica MZ16F) and were either dissected whole or micro-dissected using fine scissors and forceps.

### DNA extraction and amplicon sequencing library preparation

DNA extraction from bulk or micro-dissected tumours was performed using a QIAmp DNA FFPE Tissue kit (Qiagen, 56404) according to the manufacturer’s instructions, apart from a longer lysis incubation time of 12 h at 56 °C and omission of the 90 °C incubation step. The purified DNA was quantified using a NanoDrop spectrophotometer. Extracted DNA was stored at −20 °C.

### Targeted amplicon panel design

Standard BioTools’ D3 Assay Design software was used to design a targeted panel of primers covering ten genes (*Apc*, *Ctnnb1*, *Kras*, *Nras*, *Hras*, *Braf*, *Pten*, *Fbxw7*, *Smad4* and *Trp53*). *Apc*, *Ctnnb1*, *Kras* and *Trp53* had 100% of their exonic regions covered by the panel, whereas the coverage for the other genes was limited to previously identified hotspots on an exome hybridization panel (unpublished). All the targeted nucleotides in the panel were covered by at least two amplicons apart from *Apc* and *Pten*, which had 99.2% and 77% dual coverage, respectively.

### Targeted amplicon library preparation

The targeted amplicon library was prepared according to the Standard BioTools protocol using the 8.8.6 integrated fluidic chip (IFC) and the Juno system. In brief, each IFC allowed the highly multiplexed interrogation of 48 samples against 8 independent panels of primers, leading to the generation of 286 amplicons for each sample. The harvested amplicons from each IFC were quantified using a Bioanalyzer 2100 (Agilent) and pooled equimolarly. Sequencing was performed as paired end 150-bp reads on the Illumina platform.

### Mutation calling and filtering

FASTQ files were aligned against the Genome Reference Consortium mouse genome 39 (GRCm39)^59^ using BWA-MEM (https://github.com/lh3/bwa). Mutation calling was performed using the ampliconseq pipeline (https://github.com/crukci-bioinformatics/ampliconseq) with VarDict as variant caller and a minimum allele fraction threshold of 0.01. Variant annotation was performed using Ensembl VEP^60^. The list of called mutations was filtered to remove variants that did not pass internal noise filters. Indels were removed because of the predilection of ENU to predominantly cause single-nucleotide variants^61^. Finally, variants were retained only if they were called in at least two amplicons per sample and supported by at least five mutant reads. Given that codons 73–84 and 122–139 of *Apc* were only covered by one amplicon, variants falling in these regions were manually inspected, and only retained if they were called by at least five mutant reads and a VAF of more than 0.01. This only affected one sample (2122307_26).

### RNA isolation and library preparation

Freshly dissected tumours were immediately placed in RNAlater (Thermo Fisher, AM7021) and stored at 4 °C. RNA was extracted using an RNeasy Plus Mini Kit (Qiagen, 74136) according to the manufacturer’s instructions. Purified total RNA was quantified using a Qubit 2.0 fluorometer (Thermo Fisher), and integrity was assessed using a TapeStation 4200 (Agilent Technologies). Only samples with RNA integrity number equivalent (RINe) > 8.0 were used for sequencing. Sequencing library preparation was performed with a starting input of 100 ng RNA using the Illumina Stranded mRNA Prep Kit (Illumina, 20040534), according to the manufacturer’s instructions. The equimolarly pooled library was quantified using a Quant-it High Sensitivity fluorometer, and sizing was performed with Tapestation. Sequencing (paired end 50-bp reads) was performed on an S2 flow cell on an Illumina NovaSeq6000 platform.

### RNA-sequencing data analysis

Sequence read quality was assessed using FastQC (v0.11.9; http://www.bioinformatics.babraham.ac.uk/projects/fastqc/). Adapter content was trimmed from the reads using Trimmomatic (v0.39)^62^. Trimmed reads were aligned to GRCm39 Ensembl release 103 for quality control purposes using STAR version 2.7.7a^63^ and quality control of the aligned reads was carried out using Picard tools (v2.27.3). Gene expression quantification was carried out using Salmon (v1.9.0) against indexes generated from Gencode Mouse release M30. Differential gene expression was performed using the DESeq2 package^64^. Genes were determined to be statistically differentially expressed at an adjusted *P* value of 0.05. Gene set enrichment analysis was performed in R using the GSEA function of the clusterProfiler package (version 4.4.4)^65^. Signature scores were based on the Hallmark Pathways^66^ and published gene sets for mouse ISCs by Muñoz et al.^67^, Merloz-Suarez et al.^68^ and mouse small intestinal and colonic secretory signatures from Tomic et al.^69^. Additional published Wnt pathway and *Apc* knockout gene sets, as well as an unpublished mouse intestinal-specific *Kras*^*G12D*^ list of genes were used^70^. Mouse intestinal cell-type signatures were derived from a compendium of single-cell RNA-sequencing experiments hosted at PanglaoDB^71^. Consensus Molecular Subtyping was performed on DESeq2 vst normalized counts using gene set collection C in the MmCMS package^24^ (https://github.com/MolecularPathologyLab/MmCMS). Pathway-derived subtyping was performed using the PDSclassifier package described by Malla et al.^72^, using the default prediction probability of 0.6 as recommended (https://github.com/sidmall/PDSclassifier).

For the pseudo-bulk analysis (Extended Data Fig. 4l), bulk RNA-sequencing reads from paired major and minor clones were randomly sampled at a 2:1 ratio and compiled to create a new pseudo-sample. Differential gene expression analysis was performed between these pseudo-tumours and monoclonal tumours using the DESeq2 pipeline as described above.

### Organoid culture

Standard growth medium consisted of advanced DMEM/F12 (ADF) (Invitrogen, 12634-028) with 10 mM penicillin-streptomycin (Gibco, 15140122), 10 mM l-glutamine (Gibco, 35050-061), 10 mM HEPES (Life Technologies, 15630106), N-2 supplement (Invitrogen, 17502-048), B27-supplement (Invitrogen, 12587-010), 50 ng ml^−1^ recombinant human EGF (Peprotech, AF-100-15) and 100 ng ml^−1^ recombinant murine Noggin (Peprotech, 250-38). Dissociation medium was made with DMEM (Gibco, 11965092), 2.5% FBS, 10 mM penicillin-streptomycin, 75 U ml^−1^ collagenase IV (Worthington Biochem, LS004188), 50 μg ml^−1^ liberase TL (Roche, 5401020001) and 0.8 μg ml^−1^ DNAse I (StemCell Tech, 07900).

Micro-dissected mouse intestinal tumours were immediately placed in 200 μl dissociation medium at the time of dissection. They were incubated at 37 °C for 30 min. Next, 5 ml ice-cold ADF was added and the sample was spun at 1,200 rpm at 4 °C and supernatant aspirated. The pellet was resuspended in 5 ml ADF and spun down again. After aspirating the supernatant, the pellet was resuspended in a small volume of Cultrex Basement Membrane Extract (R&D Systems, 3433-001-R1). This was plated and left to set at 37 °C for 10 min before the addition of growth media. Organoids were grown at 37 °C, 5% CO_2_ and 21% O_2_.

### CRISPR-based editing of organoids

CRISPR-based knockout of *Apc* was performed as described by Skoufou-Papoutsaki et al.^73^. In brief, mouse small intestinal organoids were single-cell dissociated at 37 °C in TrypLE Express (Gibco, 12605010) and Y-27632 (1:1,000, Tocris, 1254) for 30 min. One-hundred thousand cells were then incubated with a Cas9 enzyme (TrueCut Cas9 Protein v2, Invitrogen, A36497) and single guide RNA (sgRNA) complex (Synthego). sgRNAs were designed using Benchling (https://www.benchling.com/) and Indelphi (https://indelphi.giffordlab.mit.edu/)^74^. Guides were designed to lead to out of frame indels and therefore, truncations at codons S96 (pre-Armadillo domain), T619 (Armadillo domain) and F1378 (MCR). The sgRNA sequences were: pre-Armadillo, CCTTCGCTCCTACGGAAGTC; Armadillo: TGTCTGGCTCCGGTAAGTGA; and MCR, TGAATACGAGCGGAGTCTCC. The cells were then transferred into a 16-well nucleovette strip (Lonza) and incubated for 10 min at room temperature. Electroporation was performed on an Amaxa 4D Nucleofector (Lonza) using the DS138 programme. After 10 min at 37 °C, cells were transferred to a 0.5 ml tube, suspended in 20 μl Cultrex and plated as described above. Once organoids were formed (usually about 7–10 days after plating), single organoids were picked using a EZGrip micropipette (CooperSurgical Fertility Solutions) under microscopic visualization. Single-picked organoids were then placed in 5 μl TrypLE Express for 10 min at room temperature, mixed with 15 μl Cultrex and replated to generate clonal organoids. DNA was extracted using the PicoPure DNA extraction kit according to the manufacturer’s instructions. PCR was performed using custom designed primers and the product Sanger sequenced. PCR primers used were: Pre_Armadillo forward, GGCAGATGGGTTCAAAGGGGTAGAG; Pre_Armadillo reverse, AAACTCCCACGCACACACAGTACTT; Arm forward, TGACTCATAGAAACAGCACTGACCCA; Arm reverse, GCATGGCTGGATTTCTCAACTACCA; MCR forward, TCAGACAACACAGGAAGCAGA; and MCR reverse, GGCCCACTCTCTCTCTTCTC. Deconvolution was performed using the ICE Synthego platform (https://ice.synthego.com/) to determine the knockout score and clonality.

### Quantitative PCR

cDNA was synthesized from 1 μg RNA using the iScript cDNA synthesis kit (Bio-Rad, 1708891). Real-time quantitative PCR for *Notum* was performed using a Taqman gene expression assay (Mm01253273_m1) according to the manufacturer’s instructions on a QuantStudio 6 (Applied Biosystems). Relative fold change in gene expression was calculated using the ${2}^{-{{\rm{\Delta \Delta }}C}_{{\rm{t}}}}$ method. All ΔΔ*C*_t_ values were normalized to the housekeeping genes *Gapdh* (Mm99999915_g1) and *Rpl37* (Mm00782745_s1).

### Haplotype transcript phasing of *Apc* truncating mutations

mRNA was isolated from micro-dissected minor tumour clones as described above. First strand cDNA synthesis was carried out using a NEB ProtoScript II First Strand cDNA synthesis kit (E6560) according to the manufacturer’s instructions. Both oligo-dT or a gene specific reverse primer (rev_3699 GCCTTTTGGCATTAGATGGA) were used.

NEB Q5 High-Fidelity DNA polymerase (M0491S), with primers hybridizing to exons 4 and 16 of *Apc* (for_261-AAAAATGTCCCTTCGCTCCT and rev_3149-CTGTGAGGGACTCTGCCTTC, respectively) was used for PCR amplification of cDNA template. For each sample a different five nucleotide barcode was incorporated onto the 5′ end of the forward primer. PCR products of each minor clone sample were first analysed by gel electrophoresis to confirm expected size distribution (several bands due to alternative splicing and Cre-mediated excision of exon 15 with a mean size of 2,585 bp), purified, quantified and then pooled in an equimolar ratio. A PacBio SMRTbell library was constructed and sequenced on a PacBio Revio SMRT Cell (25 M, 24 hr) by the Earlham Institute, Norwich, UK.

Sequenced reads were provided in unaligned BAM (uBAM) format. The reads were first converted back to FASTQ format using samtools (version 1.20). Reads were demultiplexed into individual sample FASTQ files based on their barcode sequence using a custom Python script. The FASTQ files were then aligned to the mouse reference genome (GRCm39) using the Minimap2 aligner (version 2.28) with default parameters. Genomic references for alignment were downloaded from Ensembl. The resulting BAM files were split into isoform specific BAM files using bedtools (version 2.31.1). Mutation calling was carried out on each isoform/sample bam file using Mutect2 in tumour only mode on each sample using the nf-core sarek pipeline^75^ (https://github.com/nf-core/sarek). Mutect2 was run with default parameters except the “WELLFORMEDREADFILTER” was disabled in order to avoid reads being discarded due to the intron gaps, and the “max-reads-per-alignment-start” was set to 0 to avoid downsampling. References of known SNPs and short indels were downloaded from the Mouse Genomes Project. The resulting VCF files were annotated using the Ensembl Variant Effect Predictor (VEP) tool (version 112). Annotated VCF files were converted into tabular format for further analysis using a custom R script.

To determine the error rate of long-read sequencing in this context, four samples were technically duplicated. The minimum VAF threshold for downstream filtering was chosen so that duplication of mutation calls in technical replicates was achieved. This was found to be 0.02. Additionally, mutation calls were filtered to only include nonsense mutations with ≥100 read depth.

### Whole genome sequencing and copy number analysis

Libraries for whole genome sequencing were prepared from 400 ng of genomic tumour DNA using an Illumina DNA PCR-Free Prep Tagmentation kit (20041795). Prepared libraries were sequenced on a NovaSeq6000 sequencer. Reads were aligned to the mm10 reference genome using BWA v0.7.17 and aligned reads were processed with QDNAseq v1.30.0 to generate copy number data. Read counts within 100-kb bins were normalized relative to a matched normal sample from the sample model prior to segmentation. Ploidy (defined as 2 by default) and cellularity estimation (2 × average VAF) and absolute copy number fitting were carried out using Rascal v0.7.0^76^ (https://github.com/crukci-bioinformatics/rascal).

### Confetti labelling simulation

To perform the Confetti labelling simulation (Extended Data Fig. 1f), a custom PERL script was written to randomly assign a Confetti label to crypts in a 10 × 100 field of unmarked crypts, using the observed Confetti fluorophore frequencies (CFP 2.7%, YFP 4.1%, and RFP 3.8%). The number of patches (contiguous crypts with the same fluorophore) was quantified for each simulation (a total of 1,000 simulations were run for each fluorophore), and the distribution of coloured crypts within particular patch sizes was calculated.

### Random collision modelling

Following an approach described by Novelli et al.^14^, a Poisson approximation to the binomial distribution was carried out to estimate the number of collisions expected purely by chance. In brief, this model assumes a one-dimensional arrangement of *y* crypts containing *n* adenomas each of width *x*. The number of inter-adenoma spacings that are less than *x* (that is, the number of collisions between adenomas) is approximately binomially distributed with a probability $P=1-{{\rm{e}}}^{-\frac{nx}{y}}$. From this, the expected number of collisions for each intestinal region (and therefore, heterotypic tumours) is given by *nP* and the variance by *nP*(*1* *−* *P*).

To investigate whether heterotypic tumours arise in regions of highest density, the spatstat package was used to calculate local spatial tumour density within each imaged bowel segment. The local spatial density was extracted at the location of each tumour, and densities at heterotypic tumours were compared to those of non-heterotypic tumours using a Q–Q plot and the Kolmogorov–Smirnov test. This allows assessment of whether the density distributions differ between these groups, with the hypothesis that densities would be higher on average at heterotypic tumour locations if clustering occurs in high density regions.

Additionally, a simulation making use of the observed growth rates of tumours in the *Apc*^*het*^ + ENU model was created. For each confocally imaged region of intestine, a number of points were initialized at *t* = 0 after ENU with each point attributed a Confetti label (based on observed frequencies of CFP, RFP, YFP and uncoloured crypts) and a growth rate sampled from the distribution of observed growth rates. These points were then allowed to expand until the simulation was stopped at the humane endpoint reached by the mouse. The number of collisions resulting in heterotypic tumours for each of these simulations was tallied. The simulations were repeated for 10,000 seeds for each segment of intestine scored. The observed number of heterotypic tumours was then compared to the expected number for each sample using a paired two-tailed *t*-test.

### Modelling of tumour growth

To quantify the growth dynamics of heterotypic and homotypic tumours (Fig. 4d), a mixed-effects model was built using the package nlme using mouse identity as a random effects term. The rate of tumour expansion was quantified during the exponential growth phase (that is, between timepoints 40 and 63 days after ENU).

### Bootstrap approach for *Apc* mutation probability

Inference for the comparison of the probabilities of mutation per location between the monoclonal and major (independent) groups, monoclonal and minor (independent) groups and major and minor (dependent) groups was performed by defining percentile confidence intervals via a per tumour type (that is, monoclonal versus polyclonal) stratified non-parametric bootstrap for the difference in probabilities of interest.

### Statistical analyses and reproducibility

Visualization and statistical analysis of data were performed in the R statistical computing environment (version 4.2.3) or GraphPad Prism version 10.2.2 (341). Multiple testing correction of *P* values was carried out using the Benjamini–Hochberg method^77^ for the RNA sequencing. All immunostaining and RNAscope experiments were performed on at least three independent biological replicates (three different mice). Micrographs depict representative data derived from at least three independent biological replicates. Statistical tests and corresponding *P* values are indicated in the figure legends and figures, respectively. Box plots display the distribution of data using the following components: lower whisker show the smallest observation greater than or equal to lower hinge minus 1.5× IQR; lower hinge shows the 25% quantile; the centre line shows the median, 50% quantile; the upper hinge shows the 75% quantile; the upper whisker shows the largest observation less than or equal to upper hinge plus 1.5× IQR.

### Reporting summary

Further information on research design is available in the Nature Portfolio Reporting Summary linked to this article.

## Online content

Any methods, additional references, Nature Portfolio reporting summaries, source data, extended data, supplementary information, acknowledgements, peer review information; details of author contributions and competing interests; and statements of data and code availability are available at 10.1038/s41586-024-08053-0.

## Supplementary information

Supplementary Fig. 1 This supplementary figure contains the uncropped gel relating to Extended Data Fig. 3d.

Reporting Summary

Supplementary Table 1 This table contains details for the amplicons used in the amplicon sequencing experiment.

## Source data

Source Data Fig. 1

Source Data Fig. 2

Source Data Fig. 3

Source Data Fig. 4

## Data Availability

The RNA-sequencing data generated in this study are publicly available through the Gene Expression Omnibus (GEO) with the accession code GSE272850. DNA sequencing data, including amplicon sequencing and long-read sequencing, have been deposited to the Sequence Read Archive (SRA) with BioProject ID PRJNA1141743. Source data are also available via figshare at 10.6084/m9.figshare.24771732 (ref. ^78^). Source data are provided with this paper.

## Code availability

Custom scripts to generate the bootstrap analysis and random collision modelling are available on https://github.com/sadieni/polyclonality.
