# Supplementary material for: Polyclonality overcomes fitness barriers in Apc-driven tumorigenesis
Source: Nature. 2024 Oct 30;634(8036):1196–203. doi: 10.1038/s41586-024-08053-0 (PMC11525183; doi:10.1038/s41586-024-08053-0)
Supplement: Supplementary file 1 — This supplementary figure contains the uncropped gel relating to Extended Data Fig. 3d. [file 41586_2024_8053_MOESM1_ESM.pdf]

nature portfolio

https://doi.org/10.1038/s41586-024-08053-0

Supplementary information

Polyclonality overcomes fitness barriers in *Apc*-driven tumorigenesis

In the format provided by the authors and unedited

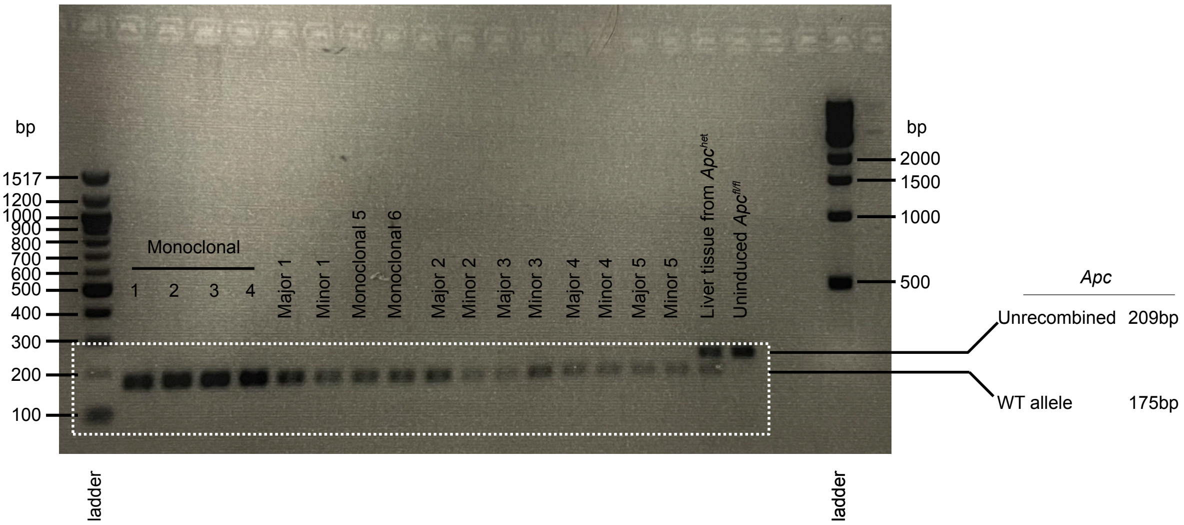

Supplementary Figure 1: Uncropped gel. Uncropped ethidium bromide stained agarose gel related to Extended Data Fig. 3d. White dashed line represents the cropped region presented in the corresponding Figure.
