# Supplementary material for: Polyclonality overcomes fitness barriers in Apc-driven tumorigenesis
Source: Nature. 2024 Oct 30;634(8036):1196–203. doi: 10.1038/s41586-024-08053-0 (PMC11525183; doi:10.1038/s41586-024-08053-0)
Supplement: Supplementary file 2 — Reporting Summary [file 41586_2024_8053_MOESM2_ESM.pdf]

Corresponding author(s): Doug Winton
Last updated by author(s): 18/8/24

Reporting Summary

Nature Portfolio wishes to improve the reproducibility of the work that we publish. This form provides structure for consistency and transparency in reporting. For further information on Nature Portfolio policies, see our Editorial Policies and the Editorial Policy Checklist.

Statistics

For all statistical analyses, confirm that the following items are present in the figure legend, table legend, main text, or Methods section.

n/a	Confirmed	
☐	☒	The exact sample size (n) for each experimental group/condition, given as a discrete number and unit of measurement
☐	☒	A statement on whether measurements were taken from distinct samples or whether the same sample was measured repeatedly
☐	☒	The statistical test(s) used AND whether they are one- or two-sided<br>*Only common tests should be described solely by name; describe more complex techniques in the Methods section.*
☒	☐	A description of all covariates tested
☐	☒	A description of any assumptions or corrections, such as tests of normality and adjustment for multiple comparisons
☐	☒	A full description of the statistical parameters including central tendency (e.g. means) or other basic estimates (e.g. regression coefficient) AND variation (e.g. standard deviation) or associated estimates of uncertainty (e.g. confidence intervals)
☐	☒	For null hypothesis testing, the test statistic (e.g. F, t, r) with confidence intervals, effect sizes, degrees of freedom and P value noted<br>*Give P values as exact values whenever suitable.*
☒	☐	For Bayesian analysis, information on the choice of priors and Markov chain Monte Carlo settings
☒	☐	For hierarchical and complex designs, identification of the appropriate level for tests and full reporting of outcomes
☒	☐	Estimates of effect sizes (e.g. Cohen's d, Pearson's r), indicating how they were calculated

Our web collection on statistics for biologists contains articles on many of the points above.

Software and code

Policy information about availability of computer code

Data collection

Microscopy and scoring:
Leica SP5 TCS confocal microscope with LAS X software (v2.8.0, Leica)
QuPath (v.0.4.3, https://github.com/qupath/qupath)
ImageJ2 (version 2.14.0/1.54f)

Quantitative PCR:
QuantStudio 6 (Applied Biosystems)

Amplicon design:
Standard Biotool D2 Design Software (https://d3.standardbio.com/account/login)

CRISPR guide design:
Benchling (https://www.benchling.com/)
Indelphi (https://indelphi.giffordlab.mit.edu/)

CRISPR analysis:
ICE Synthego platform (https://ice.synthego.com/)

Custom code deposited at: https://github.com/sadieni/polyclonality

Data analysis

Analyses were performed predominantly with R version 4.2.3 (2023-03-15) or GraphPad Prism version 10.2.2 (341).

Data analysis

RNA sequencing analysis:
FastQC (v0.11.9; http://www.bioinformatics.babraham.ac.uk/projects/fastqc/)
Trimmomatic (v0.39)
STAR version 2.7.7a
Picard tools (v2.27.3)
Salmon (v1.9.0)
DESeq2 (version 3.19)
clusterProfiler package (version 4.4.4)
MmCMS package24 (https://github.com/MolecularPathologyLab/MmCMS)
PDSclassifier package (https://github.com/sidmall/PDSclassifier)

Mutational analysis:
BWA-MEM (https://github.com/lh3/bwa)
ampliconseq pipeline (https://github.com/crukci-bioinformatics/ampliconseq)

Long read sequencing:
Minimap2 aligner (version 2.28)
nf-core sarek pipeline (https://github.com/nf-core/sarek)
bedtools (version 2.31.1)

Whole genome sequencing:
BWA v0.7.17
QDNAseq v1.30.0
Rascal v0.7.0 (https://github.com/crukci-bioinformatics/rascal)

For manuscripts utilizing custom algorithms or software that are central to the research but not yet described in published literature, software must be made available to editors and reviewers. We strongly encourage code deposition in a community repository (e.g. GitHub). See the Nature Portfolio guidelines for submitting code & software for further information.

Data

Policy information about availability of data

All manuscripts must include a data availability statement. This statement should provide the following information, where applicable:

- Accession codes, unique identifiers, or web links for publicly available datasets
- A description of any restrictions on data availability
- For clinical datasets or third party data, please ensure that the statement adheres to our policy

The RNA sequencing data generated in this study are publicly available through the Gene Expression Omnibus (GEO) with the accession code GSE272850. DNA sequencing data, including amplicon sequencing and long-read sequencing, have been deposited to the Sequence Read Archive (SRA) with BioProject ID PRJNA1141743. Source data is provided with this manuscript and is also available via figshare at https://doi.org/10.6084/m9.figshare.24771732.

Research involving human participants, their data, or biological material

Policy information about studies with human participants or human data. See also policy information about sex, gender (identity/presentation), and sexual orientation and race, ethnicity and racism.

Reporting on sex and gender	N/A
Reporting on race, ethnicity, or other socially relevant groupings	N/A
Population characteristics	N/A
Recruitment	N/A
Ethics oversight	N/A

Note that full information on the approval of the study protocol must also be provided in the manuscript.

Field-specific reporting

Please select the one below that is the best fit for your research. If you are not sure, read the appropriate sections before making your selection.

☒ Life sciences ☐ Behavioural & social sciences ☐ Ecological, evolutionary & environmental sciences

For a reference copy of the document with all sections, see nature.com/documents/nr-reporting-summary-flat.pdf

Life sciences study design

All studies must disclose on these points even when the disclosure is negative.

Sample size	Formal sample size and power analysis were not performed due to the lack of extensive a priori data and uncertain estimates of effect size with this newly described model. Instead, the sample sizes took into consideration preliminary effect sizes from pilot experiments and balanced the 3Rs (Replacement, Reduction, Refinement) to limit the unnecessary use of animals.
Data exclusions	No data were excluded, unless mentioned otherwise.
Replication	Tissue sections from 3 independent animals (n=3) were stained for all immunohistochemistry, immunofluorescence and in-situ hybridisation. For the organoid experiments, 3 independent passages from one biological replicate for each of the three knockout lines generated were used. All attempts at replication were successful.
Randomization	Control and experimental animals were co-housed independent of genotype and cohorts were comprised of a balance of both male and female animals. In order to reduce the impact of covariates such as gender or housing, animals were recruited to treatment groups in a partially randomised manner while taking these factors into account.
Blinding	For animal welfare reasons, researchers were not blinded to genotype during study and data collection.

Reporting for specific materials, systems and methods

We require information from authors about some types of materials, experimental systems and methods used in many studies. Here, indicate whether each material, system or method listed is relevant to your study. If you are not sure if a list item applies to your research, read the appropriate section before selecting a response.

Materials & experimental systems

n/a	Involved in the study	
☐	☒	Antibodies
☒	☐	Eukaryotic cell lines
☒	☐	Palaeontology and archaeology
☐	☒	Animals and other organisms
☒	☐	Clinical data
☒	☐	Dual use research of concern
☒	☐	Plants

Methods

n/a	Involved in the study	
☒	☐	ChIP-seq
☒	☐	Flow cytometry
☒	☐	MRI-based neuroimaging

Antibodies

Antibodies used

Antibody, catalog number and dilutions used for immunohistochemistry or immunofluorescence included in the manuscript.
β-catenin (0.25 ug/ml, mouse, 610154, BD Biosciences)
O-6-ethyl-guanine (0.5 ug/ml, rat, SQX-SQM001, Squarix Biotechnology)
RFP (1:100, rabbit, R10367, Thermo Fisher)
GFP (1:100, chicken, ab13970, Abcam)
Lysozyme (1:100, goat, sc-27958, Santa Cruz)
UEA-1 (AbD Serotec, 9420-00024)
Ki67 (1:100, rat, 14-5698-82, Thermo Fisher)

Secondary antibodies:
Rabbit Anti-rat, (Bethyl Laboratories, A110-322A) at 1:250
Rabbit Anti-mouse IgG1 (Abcam, ab125913) at 1:1500
Donkey anti-rabbit (ThermoFisher, A31572) at 1:200
Goat anti-chicken (ThermoFisher, A11039) at 1:200
Donkey anti-goat (ThermoFisher, A21447) at 1:200

Validation

1. β-catenin (0.25 ug/ml, mouse, 610154, BD Biosciences); used for IHC. Species Reactivity: Human, Mouse, Rat, Dog, Chicken. This antibody was validated by the company and used in our previous study (PMID: 33093165).
2. O-6-ethyl-guanine (0.5 ug/ml, rat, SQX-SQM001, Squarix Biotechnology); used for IHC. Species Reactivity: Human, Mouse, Rat. This antibody was validated by the company and used in a previous study (PMID: 29958939).
3. RFP (1:100, rabbit, R10367, Thermo Fisher); used for IHC, IF. Species independent. This antibody was validated by the company.
4. GFP (1:100, chicken, ab13970, Abcam); used for IHC and IF, WB. Species independent. This antibody was validated by the company.
5. Lysozyme (1:100, goat, sc-27958, Santa Cruz); used for IF Species reactivity: Human, Mouse. This antibody was validated by the company.
6. UEA-1 (AbD Serotec, 9420-00024). Validated in house (https://www.ncbi.nlm.nih.gov/pmc/articles/PMC6138952/).

	7. Ki67 (14-5698-82, Thermo Fisher). Used for IF. Species reactivity: Dog, Cynomolgus monkey, Human, Mouse, Non-human primate, Rat. This antibody was validated by the company.

Animals and other research organisms

Policy information about studies involving animals; ARRIVE guidelines recommended for reporting animal research, and Sex and Gender in Research

Laboratory animals	Mice of both sexes aged at least 8 weeks old were induced. C57/BL6J mice with a combination of Villin-CreERT2, Apcfl/+, R26R-Confetti, LSL-KrasG12D, or Trp53fl/fl alleles were used.
Wild animals	No wild animals were used in the study.
Reporting on sex	Both male and female mice were used in all experiments.
Field-collected samples	No field-collected samples were used in this study.
Ethics oversight	All animal experiments were performed in accordance with UK Home Office regulations (under project licence PD5F099BE). They adhered to the ARRIVE guidelines and were subject to review by the Animal Welfare and Ethical Review Board of the CRUK Cambridge Institute.

Note that full information on the approval of the study protocol must also be provided in the manuscript.

Plants

Seed stocks	*Report on the source of all seed stocks or other plant material used. If applicable, state the seed stock centre and catalogue number. If plant specimens were collected from the field, describe the collection location, date and sampling procedures.*
Novel plant genotypes	*Describe the methods by which all novel plant genotypes were produced. This includes those generated by transgenic approaches, gene editing, chemical/radiation-based mutagenesis and hybridization. For transgenic lines, describe the transformation method, the number of independent lines analyzed and the generation upon which experiments were performed. For gene-edited lines, describe the editor used, the endogenous sequence targeted for editing, the targeting guide RNA sequence (if applicable) and how the editor was applied.*
Authentication	*Describe any authentication procedures for each seed stock used or novel genotype generated. Describe any experiments used to assess the effect of a mutation and, where applicable, how potential secondary effects (e.g. second site T-DNA insertions, mosiacism, off-target gene editing) were examined.*
